# Building a genomic framework for prospective MRSA surveillance in the United Kingdom and the Republic of Ireland

**DOI:** 10.1101/gr.196709.115

**Published:** 2016-02

**Authors:** Sandra Reuter, M. Estée Török, Matthew T.G. Holden, Rosy Reynolds, Kathy E. Raven, Beth Blane, Tjibbe Donker, Stephen D. Bentley, David M. Aanensen, Hajo Grundmann, Edward J. Feil, Brian G. Spratt, Julian Parkhill, Sharon J. Peacock

**Affiliations:** 1 Department of Medicine, University of Cambridge, Cambridge CB2 0QQ, United Kingdom;; 2 Pathogen Genomics, Wellcome Trust Sanger Institute, Hinxton CB10 1SA, United Kingdom;; 3 Public Health England, Microbiology Services Division, Addenbrooke’s Hospital, Cambridge CB2 0QW, United Kingdom;; 4 Cambridge University Hospitals NHS Foundation Trust, Cambridge CB2 0QQ, United Kingdom;; 5 School of Medicine, University of St. Andrews, St. Andrews KY16 9TF, United Kingdom;; 6 British Society for Antimicrobial Chemotherapy, B1 3NJ, United Kingdom;; 7 North Bristol NHS Trust, Bristol BS10 5NB, United Kingdom;; 8 Department of Medical Microbiology, University Medical Centre Groningen, Rijksuniversiteit Groningen, 9713 GZ Groningen, The Netherlands;; 9 Faculty of Medicine, School of Public Health, Imperial College, London W2 1PG, United Kingdom;; 10 Department of Hospital Epidemiology, Institute for Environmental Medicine and Hospital Hygiene, University Hospital Freiburg, 79106 Freiburg, Germany;; 11 The Milner Centre for Evolution, Department of Biology and Biochemistry, University of Bath, Bath BA2 7AY, United Kingdom;; 12 London School of Hygiene and Tropical Medicine, London, WC1E 7HT, United Kingdom

## Abstract

The correct interpretation of microbial sequencing data applied to surveillance and outbreak investigation depends on accessible genomic databases to provide vital genetic context. Our aim was to construct and describe a United Kingdom MRSA database containing over 1000 methicillin-resistant *Staphylococcus aureus* (MRSA) genomes drawn from England, Northern Ireland, Wales, Scotland, and the Republic of Ireland over a decade. We sequenced 1013 MRSA submitted to the British Society for Antimicrobial Chemotherapy by 46 laboratories between 2001 and 2010. Each isolate was assigned to a regional healthcare referral network in England and was otherwise grouped based on country of origin. Phylogenetic reconstructions were used to contextualize MRSA outbreak investigations and to detect the spread of resistance. The majority of isolates (*n* = 783, 77%) belonged to CC22, which contains the dominant United Kingdom epidemic clone (EMRSA-15). There was marked geographic structuring of EMRSA-15, consistent with widespread dissemination prior to the sampling decade followed by local diversification. The addition of MRSA genomes from two outbreaks and one pseudo-outbreak demonstrated the certainty with which outbreaks could be confirmed or refuted. We identified local and regional differences in antibiotic resistance profiles, with examples of local expansion, as well as widespread circulation of mobile genetic elements across the bacterial population. We have generated a resource for the future surveillance and outbreak investigation of MRSA in the United Kingdom and Ireland and have shown the value of this during outbreak investigation and tracking of antimicrobial resistance.

Methicillin-resistant *Staphylococcus aureus* (MRSA) was first isolated in 1961 in the United Kingdom (UK), 1 yr after methicillin was introduced into clinical practice (Jevons 1961). The prevalence of MRSA gradually increased thereafter, and by 1971, 5% of *S. aureus* isolates referred to the National Staphylococcal Reference Laboratory were MRSA (Marples and Reith 1992). Outbreaks of gentamicin-resistant MRSA in several hospitals during the late 1970s (Shanson 1981) were followed by the emergence of MRSA with potential for epidemic spread (Johnson et al. 2005). By the mid-1980s, MRSA had spread across the UK, and the majority were epidemic (E)MRSA-1, later assigned as sequence type (ST) 239 by multilocus sequence typing (MLST) (Kerr et al. 1990; Johnson et al. 2005). A decline in EMRSA-1 in the late 1980s and early 1990s was associated with an increase in EMRSA-3 (ST 5) (Marples and Reith 1992; Richardson and Reith 1993; Cox et al. 1995; Enright et al. 2002). This dynamic process continued with the emergence in the early 1990s of EMRSA-15 (ST 22) and EMRSA-16 (ST 36) (Richardson and Reith 1993; Cox et al. 1995; Enright et al. 2002), which disseminated across the UK. These two clones continue to predominate, with EMRSA-15 accounting for ∼85% of MRSA bloodstream infections in the UK in 2007 and with trends suggesting that EMRSA-16 is in decline (Ellington et al. 2010; McAdam et al. 2012). Antimicrobial resistance is known to differ between EMRSA-15 and -16, with EMRSA-16 being the more resistant lineage of the two. However, for both lineages, the acquisition of the SCC*mec* element conferring methicillin resistance and the presence of mutations in *gyrA* and *grlA* conferring fluoroquinolone resistance are considered to be major contributors to the success of these epidemic lineages (Knight et al. 2012; McAdam et al. 2012; Holden et al. 2013).

Bacterial genotyping using pulsed-field gel electrophoresis (PFGE), MLST, and *spa* typing has been used to identify epidemic clones and to give insights into the microevolutionary dynamics of predominant MRSA lineages in the UK. However, these methods have limited resolution and lack discriminatory power when one or a small number of clones predominate (McAdam et al. 2012; Miller et al. 2014; Bartels et al. 2015). This means that once widely established, the subsequent dynamics of clonal MRSA spread within and between healthcare facilities cannot be fully elucidated. As a result, bacterial typing does not form a central component of MRSA transmission and outbreak investigation. Several recent publications have confirmed the ability of whole-genome sequencing (WGS) to define transmission dynamics of a single clone at different geographic and temporal scales. This has identified global and local transmission routes and, when combined with epidemiological data, can confirm or refute putative MRSA outbreaks (Köser et al. 2012; Harris et al. 2013; Nubel et al. 2013; Miller et al. 2014; Török et al. 2014; Bartels et al. 2015). Similarly, while surveillance of MRSA has been carried over several years and a limited number of point prevalence studies of variable methodology have been undertaken in different settings, serial systematic prevalence studies of individual epidemic lineages are lacking (Johnson et al. 2012; Afshinnekoo et al. 2015; Bartels et al. 2015; Peng et al. 2015). WGS could potentially be used for national and local surveillance of MRSA lineages and to enhance the investigation of suspected outbreaks, but comprehensive genomic databases are required to provide the context that would allow robust epidemiological interferences. Here, we describe the analysis of over 1000 MRSA genomes drawn from across the UK and Ireland over a period of a decade and the first evaluation of this rich data set to describe the macroepidemiology of MRSA.

## Results

### Sampling framework for MRSA genome database

**Figure 1.**
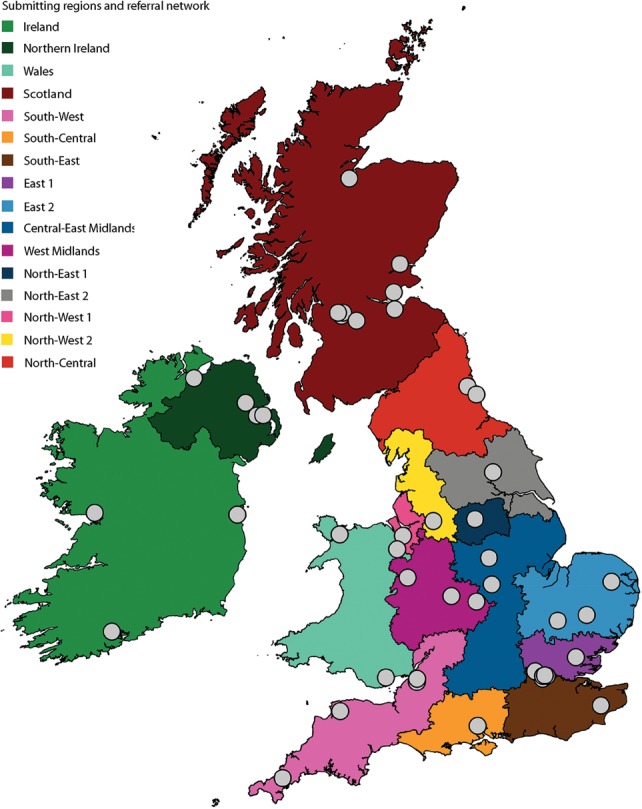
Map of laboratories, submitting regions, and referral networks. Laboratories submitting isolates to the British Society of Antimicrobial Chemotherapy collection are shown as a gray dot. Regions are colored as indicated, and English regions were based on referral networks as previously described (Donker et al. 2014).

Our first objective was to define the population structure and dynamics of MRSA across England, Northern Ireland, Wales, Scotland (together making up the UK), and the Republic of Ireland over the last decade using WGS. This was achieved by partnering with the British Society for Antimicrobial Chemotherapy (BSAC), which coordinates an antimicrobial resistance surveillance project across this geographical area (for details, see www.bsacsurv.org) (Reynolds et al. 2008). The sampling framework is systematic and unbiased since diagnostic microbiology laboratories submit a defined number of consecutive but nonduplicate *S. aureus* isolated from blood cultures each year. A total of 47 centers contributed *S. aureus* bacteremia isolates to the BSAC Bacteremia Resistance Surveillance Program between 2001 and 2010, 24–25 centers each year from 2001–2009, and 38 in 2010 when the network was expanded. Sixteen centers contributed in all 10 yr; 13 contributed only in 2010. The BSAC target was to receive 10 *S. aureus* isolates per center per year in 2001–2007, 20 in 2008–2009, and 14 in 2010. Actual collections were 97% of target on average, and <5% of 261 center-year collections were <90% complete. MRSA bacteremia isolates were confirmed and sequenced from the 45 laboratories between 2001 and 2010, the geographic locations of which are shown in Figure 1 (for details, see Supplemental Table S1). The number of MRSA isolates sequenced by year is shown in Supplemental Figure S1.

Regional healthcare referral networks have been described for England. These consist of hospitals in geographical proximity that frequently exchange patients through referrals and are defined by boundaries beyond which there is a sharp fall in patient movement (Donker et al. 2014). Patient transfer between hospitals is highly relevant to patterns of MRSA transmission, and so we placed our phylogenetic analysis in the context of these referral networks. Each network contained at least one submitting laboratory, with some networks containing multiple laboratories (Fig. 1; Supplemental Table S1). Around 100 MRSA samples were sequenced per year, with an average of six isolates per year per network. Published data are not available on referral networks within Wales, Scotland, Northern Ireland, and the Republic of Ireland, and these were each treated as single entities for the purposes of the analysis (Fig. 1; Supplemental Table S1).

### Overview of the population structure of MRSA in the UK

The frequency of each clonal complex (CC) by year is shown in Table 1. CCs are STs that share five or more alleles across the seven loci examined (Cooper and Feil 2004). The majority of isolates (*n* = 783, 77%) belonged to CC22, which contains the dominant epidemic clone in the UK and Ireland, EMRSA-15. The second most frequent CC was CC30 (*n* = 144, 14%), containing the epidemic clone EMRSA-16 (ST36). Isolates in CC1, CC5, and CC8 each made up between 1% and 3% of the population, with <1% of isolates assigned to CC9, -15, -25, -45, -59, and -97, as well as previously undefined CCs. Just two isolates were found to belong to the USA300 clone, which is the most dominant MRSA clone in the United States of America (Uhlemann et al. 2014). Of the isolates submitted, only three CCs—CC22, CC30, and CC5—had been retrieved continuously in every year of the study period, whereas all other CCs were found sporadically. These data describe a period of clonal stability with no evidence for newly emerging lineages. Subsequent analyses of the MRSA populations were lineage specific and examined the fine-scale variation within the two major lineages.

**Table 1.**
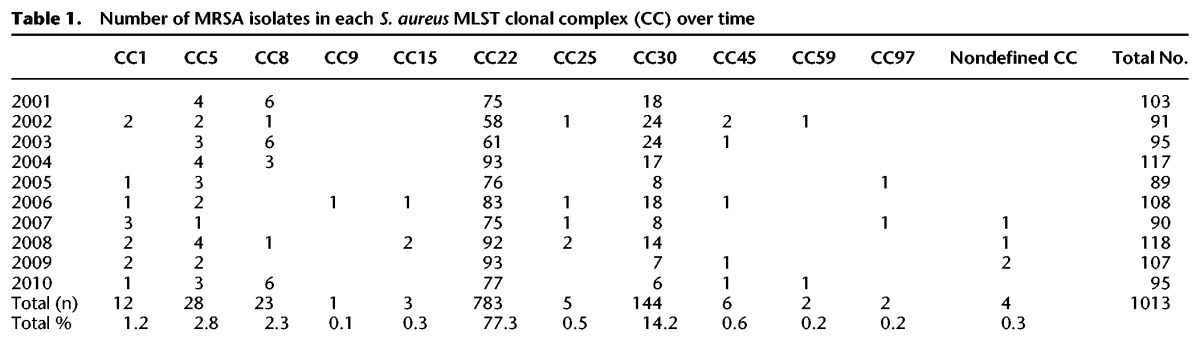
Number of MRSA isolates in each *S. aureus* MLST clonal complex (CC) over time

### Lineage-specific phylogenetic analyses

**Figure 2.**
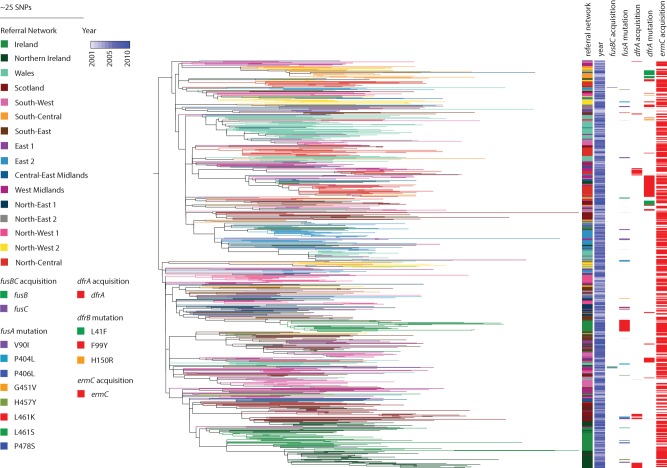
Phylogeny of EMRSA-15 and distribution of antibiotic resistance genes. The maximum likelihood tree of ST22-A isolates was based on core SNPs identified by mapping to the reference ST22 genome HO 5095 0412. The total number of SNP sites present in the core genome alignment in this lineage was 20,488 SNPs. Branches are labeled with colors of the referral networks as indicated by the legend. Represented is the year of isolation, as well as the acquisition of *fusB* or *fusC*, *fusA* mutations, acquisition of *dfrA*, and *dfrA* mutations, and *ermC* acquisition as indicated by the legend.

Phylogenetic reconstruction based on 21,848 core SNPs within the 783 CC22 isolates (Supplemental Figs. S2, S3) revealed a tightly related, hospital-adapted lineage containing EMRSA-15 (ST22-A2) and its progenitor lineage (non-ST22-A1) and a genetically more divergent community-associated population (non-ST22-A) as described previously (Holden et al. 2013). Therefore we reconstructed the phylogeny focusing only on EMRSA-15, which gave 20,488 core SNP sites in 775 isolates (Fig. 2; Supplemental Fig. S4). Genome-based studies of global clones of MRSA (e.g., ST239 [Harris et al. 2010], ST22 [Holden et al. 2013]) have demonstrated genetic clustering of isolates according to country/continent of isolation, and therefore, we hypothesized that EMRSA-15 would demonstrate geographical clustering in the UK and Ireland. By color-coding each isolate in the phylogeny according to the referral network, we showed the presence of strong phylogenetic clustering by referral network (Fig. 2). Based on all internal nodes in the phylogeny of isolates from England, quantitative analysis revealed that 44.1% of all internal nodes had descendants from strictly the same referral network, thus clustering within their respective originating network or region. To substantiate this hypothesis, isolates were randomly assigned to referral network locations. The average of these 1000 permutation tests showed on average only 3.0% (IQR: 2.4%–3.6%) of isolates clustered with isolates within their referral network, indicating that the strong geographic signal is not arbitrary.

Study isolates were drawn from a period of time that post-dates the dissemination of EMRSA-15 across the UK and Ireland, and the sequence data generated here are consistent with regional diversification following widespread dissemination. In some instances, EMRSA-15 appears to have been introduced into a region on several occasions, as reflected by the existence of several distinct clades (e.g., in referral networks South-West and North-Central) (Fig. 2). We also observed that an introduction event was sometimes followed by this lineage becoming established and coexisting with other distinct clades, for example, in North-Central.

**Figure 3.**
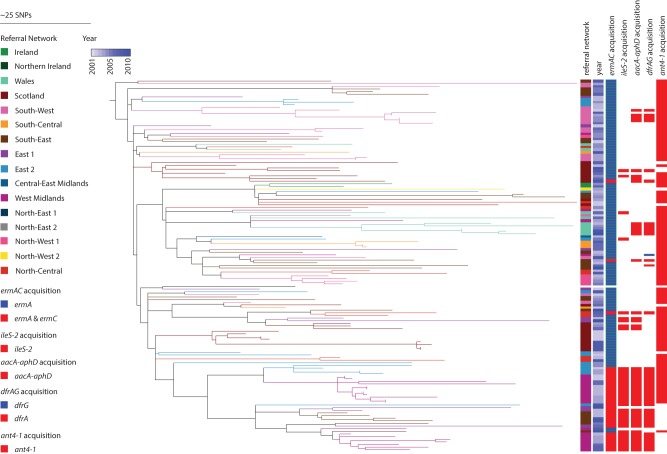
Phylogeny of EMRSA-16 and distribution of antibiotic resistance genes. Maximum likelihood tree based on core SNPs identified by mapping to the reference ST36 genome MRSA252. The total number of SNP sites present in the core genome alignment in this lineage was 4400 SNP sites. Branches are labeled with colors of the referral networks as indicated by the legend. Represented is the year of isolation, as well as acquisition of *ermA* and *ermC* (erythromycin resistance), *ileS-2* (mupirocin resistance), *aacA-aphD* (aminoglycoside resistance), *dfrA* and *dfrG* (trimethoprim resistance), and *ant4-1* (aminoglycoside resistance), as indicated by the legend.

A phylogenetic tree based on 4400 core SNP sites for 140 EMRSA-16 (ST36) isolates revealed a similar structure to the EMRSA-15 (ST22-A) phylogeny. Although EMRSA-16 was more sparsely populated, it was widely disseminated with evidence of geographic structuring (Fig. 3), as reported previously (McAdam et al. 2012). Analysis of less common CCs suggested some clustering; for example, eight of 12 CC1 isolates were from the South-West, and 11 of the 28 CC5 isolates were from Wales.

### Using the genomic framework to contextualize outbreaks

**Figure 4.**
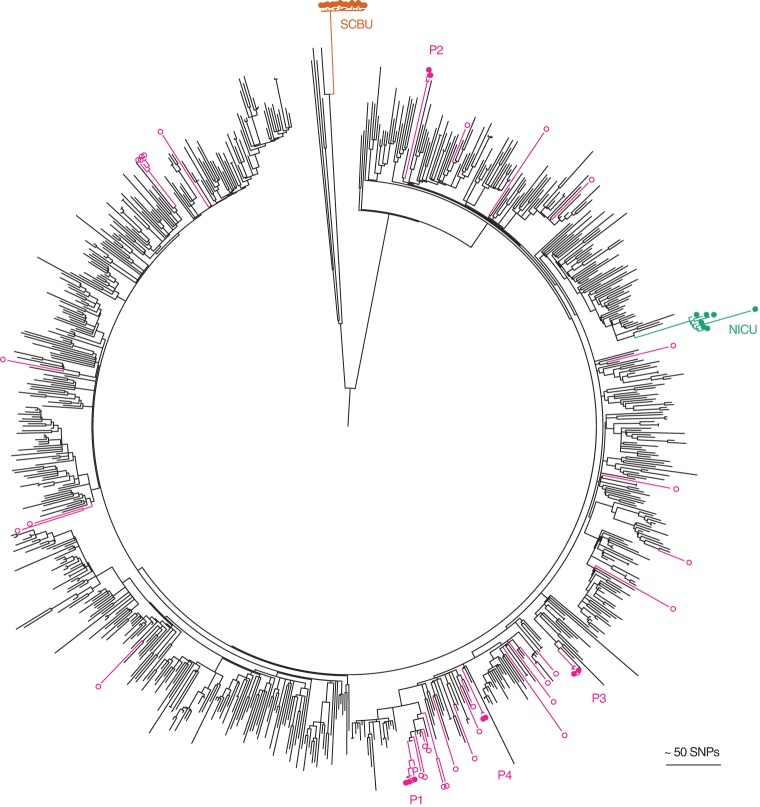
Contextualization of outbreak investigations of CC22 MRSA studied at Cambridge University Hospitals. The maximum likelihood tree was based on 22,238 core SNPs for the 783 ST22 genomes, together with seven isolates from an MRSA outbreak on a neonatal intensive care unit (NICU, green) (Köser et al. 2012), 15 isolates from an MRSA outbreak that focused on a special care baby unit (SCBU, orange) but extended to other wards and the community (Harris et al. 2013), and 42 isolates sequenced as part of an MRSA outbreak investigation on a hepatology ward (nine isolates from four patients with bacteremia [P1-4; pink filled dots] and the remainder from patients who were MRSA carriers on the same ward during a comparable timeframe [pink open dots]) (Török et al. 2014).

Next, we tested the utility of this genetic database for the investigation of suspected MRSA outbreaks. We have described previously the use of WGS to both confirm and refute local outbreaks at the Cambridge University Hospitals NHS Foundation Trust (Köser et al. 2012; Harris et al. 2013; Török et al. 2014), and we placed these within the context provided by the national BSAC collection. The first MRSA outbreak involved seven infants in a single neonatal intensive care unit (NICU) caused by EMRSA-15, in which one isolate in the cluster was genetically divergent compared with the other isolates as a result of being a hypermutator (Köser et al. 2012). The second MRSA outbreak initially involved a special care baby unit (SCBU), but MRSA was shown through genetic analysis to have spread to mothers on a post-natal ward, to 10 patients in the community who developed skin and soft tissue infections, and to a colonized healthcare worker in the SCBU (Harris et al. 2013). The outbreak MRSA was a novel ST (ST2371), which is highly related to ST22 (a single locus variant). A third investigation of four patients with five episodes of MRSA who had overlapping periods of admission on a hepatology ward showed that they had been infected by their carriage strain and thus refuted an outbreak on the ward (Török et al. 2014). Placing genomes from these three investigations into the phylogenetic tree of the 783 CC22 isolates demonstrated striking demarcation for the two outbreak clusters but scattering throughout the tree for MRSA from the hepatology pseudo-outbreak. This analysis also demonstrated that the two outbreaks in units contained within the same hospital building were caused by phylogenetically distant lineages (Fig. 4).

### Determining distribution and spread of resistance at different regional levels

**Figure 5.**
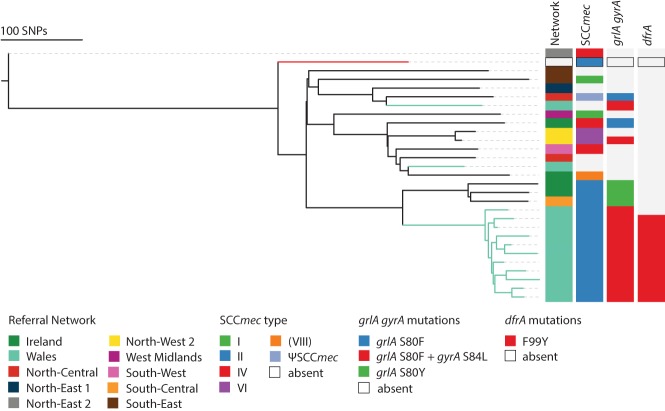
Phylogeny of CC5. The maximum likelihood tree was based on core SNPs identified by mapping to the reference ST5 genome N315. The total number of SNP sites present in the core genome alignment in this lineage was 3229 SNP sites. Welsh isolates have light green branches, and the branch leading to reference N315 is colored red.

The database could also be used for the surveillance of antimicrobial resistance in MRSA. To test this, we mined the BSAC data set to illustrate the potential to highlight (1) local expansion of resistance, (2) regional expansion of a specific resistance determinant, and (3) resistance determinants that are more widely disseminated. Localized emergence of antimicrobial resistance was observed for isolates in a closely related lineage (pairwise SNP difference of 28–114) belonging to CC5 from a healthcare institution in Wales, which were clearly separate from the rest of the CC5 isolates (Fig. 5). These 11 isolates all contain the Tn*554* transposon encoding *ermA* for erythromycin and clindamycin resistance, as well as type II staphylococcal cassette chromosome *mec* element encoding methicillin resistance (SCC*mec* II). Further antibiotic resistance mutations were apparent in this cluster with the presence of both *grlA* and *gyrA* mutations for fluoroquinolone resistance. Additionally, 10 out of 11 isolates also contained a mutation in *dfrA* conferring trimethoprim resistance. This extended spectrum of antibiotic resistance may explain their apparent regional success in a single healthcare institution.

We noted that EMRSA-15 isolates from the Republic of Ireland had a higher prevalence of fusidic acid resistance compared with isolates from the UK (37.5% vs. 10.4%, respectively). A high prevalence of fusidic acid resistance has been noted previously for Ireland and Greece (Castanheira et al. 2010). Fusidic acid resistance can be conferred through gene acquisition (*fusB* or *fusC*) or by chromosomal mutations (*fusA*). Genomic analysis readily demonstrated that mutation dominated over gene acquisition as the basis for resistance (53 vs. four events, respectively) (Fig. 2). Resistance in Irish isolates arose through a single L461K mutation in *fusA* prior to expansion of this clade and can be found in several contributing centers. Two other Irish clades, found in the same hospitals over the same time period, only showed sporadic acquisition of resistance as singular, independent events (Fig. 2).

Examples of more widely distributed resistance profiles included trimethoprim resistance in EMRSA-15 and gentamicin/mupirocin resistance in EMRSA-16. Acquisition of gentamicin (*aacA-aphD*), mupirocin (*ileS-2*), erythromycin (*ermC*), and aminoglycoside (*ant4-1*) resistance genes were found to coincide with each other as well as with plasmid-related genes in EMRSA-16 (Fig. 3). The presence of these clustered genes in several referral networks (West Midlands, South-East) suggests a number of circulating plasmids in the EMRSA-16 population. Similarly, in EMRSA-15, acquisition of *dfrA* was identified to be the genetic basis for trimethoprim resistance in isolates from Northern Ireland (10 isolates), Scotland (seven isolates), and West Midlands/North-Central (four and five isolates, respectively) (Fig. 2). This is consistent with the carriage of this gene on a mobile genetic element, but since *dfrA* was found separately on its own small contig, we cannot deduce whether it was carried on a plasmid or transposon. Trimethoprim resistance was also encoded by chromosomal mutations in *dfrA*, with L41F and F99Y being prominent mutations basal to a number of referral network clusters (Fig. 2).

We also observed that the extent of antibiotic resistance within a lineage is governed by the mobile element on which it is carried, an example of which is erythromycin resistance. Contrary to other resistance determinants that appeared to be locally restricted, erythromycin resistance was very unstable within the EMRSA-15 population. This is likely due to the fact that the resistance determinant *ermC* is carried on a small 2-kb plasmid, which is readily lost and gained. In contrast, erythromycin resistance is ubiquitous in the EMRSA-16 lineage as *ermA* is carried on transposon Tn*554*, which stably integrates into the chromosome as part of the SCC*mec* II element.

## Discussion

This evaluation of over 1000 MRSA genomes representing isolates from the UK and Republic of Ireland describes the genetic structure of dominant lineages (EMRSA-15 and EMRSA-16) over the last decade. Parallels were observed between the dominant epidemic clone EMRSA-15 and the declining epidemic clone EMRSA-16, including geographic structuring and clonality. Numerous other MRSA lineages were identified at low prevalence, which mostly lacked specific phylogenetic structuring and showed no evidence of expansion over time. There was strong evidence for region-specific subclones of EMRSA-15, which at a finer level echoes a previous finding of regional and country-specific clustering for this lineage (Holden et al. 2013). Regional clustering was also observed for EMRSA-16, as was previously described (McAdam et al. 2012; Miller et al. 2014). Owing to the lack of network data for Scotland, Ireland, Wales, and Northern Ireland, it was not possible to undertake any further regional stratification within these countries. However, for the English referral network, isolates from a specific referral network were more likely to cluster with isolates from the same region than with isolates from a different network. This could be influenced by the submitting hospitals, since not all hospitals contributed MRSA isolates in every year. The current sampling strategy did, however, achieve good temporal and regional coverage. We also observed the presence of multiple subclones within individual referral networks. This is consistent with a model in which the initial UK-wide dissemination of EMRSA-15 included several introductions into each location with subsequent local diversification.

Local and regional structuring was also observed for antibiotic resistance determinants, with both epidemic clones showing regional clades with specific antibiotic resistance patterns. Country- or region-specific antibiotic treatment regimens impact the evolution of drug resistance. This has been exemplified previously for clindamycin in EMRSA-15, where Germany shows higher prescription levels than the UK and where resistance levels are significantly higher (Holden et al. 2013). Differences in antimicrobial resistance have also been noted for the USA-300 clone that is successful in the United States, with the coexistence of fluoroquinolone-resistant and -susceptible lineages (Alam et al. 2015). Here, we provide evidence for higher levels of resistance to fusidic acid in Ireland compared with the UK. Antimicrobial prescription policies or usage may differ between the UK and the Republic of Ireland, exerting a potential selective pressure leading to higher prevalence of fusidic acid resistance in Irish isolates. Locally successful clones may also differ in antibiotic resistance patterns, such as the expansion of CC5 isolates in a single healthcare institution, or distribution of trimethoprim resistance within referral networks. Wider dissemination of antibiotic resistance determinants can also be facilitated through carriage of multiple plasmids, as shown by linked gentamicin/mupirocin resistance putatively carried on plasmids in the EMRSA-16 population. The fluidity of antimicrobial resistance markers such as *ermC* in EMRSA-15 affects clinical practice, as the pattern of antimicrobial resistance is often used during infection control investigations as a proxy to determine whether isolates are related and therefore might be part of an outbreak. This was demonstrated during an outbreak on SCBU, where the *ermC* plasmid was lost in some patients (Harris et al. 2013). By looking at a wider range of isolates within geographically defined regions, it becomes possible to distinguish between widespread resistance markers and locally emerging strains, as well as the stability of mobile genetic elements within a population.

This sequence data set has considerable long-term value, since it provides a genomic framework for the surveillance of MRSA lineages and tracking of MRSA transmission between referral networks in the UK, as well as globally circulating lineages like EMRSA-15. The likely introduction of bacterial genome sequencing to confirm or refute MRSA outbreaks will need to use such data as a source of reference genomes and region-specific comparators, and the availability of this data set in public databases will provide the ability to visualize such comparisons. Equally, it can also be used to discern differences within regional clones relating to antibiotic resistance and will provide valuable information on resistance determinants as well as their horizontal propagation and targeted patient care.

## Methods

### Isolates, sequencing, and analysis

A total of 1013 MRSA isolates were provided by the BSAC bacteremia resistance surveillance program (Reynolds et al. 2008) from 2001–2010 (Supplemental Table S2; www.bsacsurv.org). DNA extraction was carried out on a QIAxtractor (Qiagen), and library preparation was performed as previously described (Köser et al. 2012). Index-tagged libraries were created, and 96 separated libraries were sequenced in each of eight channels using the Illumina HiSeq platform (Illumina) to generate 100-bp paired-end reads at the Wellcome Trust Sanger Institute. Average sequence depth was 82.32×, and average sequence quality was 39. The ST and CC of each isolate were determined from the sequence (Enright et al. 2000; Cooper and Feil 2004). Sequence reads were then mapped to relevant reference genomes based on the MLST assignment to identify single-nucleotide polymorphisms (SNPs) (Harris et al. 2010). The references genomes used were HO 5096 0412 (Köser et al. 2012) for CC 22 isolates, MRSA252 (Holden et al. 2004) for CC30 isolates, and N315 (Kuroda et al. 2001) for CC5 isolates. SNPs were identified in the derived whole-genome alignments as previously described (Holden et al. 2013), and mobile genetic elements, phage, and repetitive regions were excluded, leaving a core genome size of 2,655,809 bp. Phylogenetic estimation was done using RAxML with the general time reversal model and gamma correction (Stamatakis et al. 2005). Trees were visualized using FigTree (http://tree.bio.ed.ac.uk/software/figtree/) and iTOL (Letunic and Bork 2011). De novo assembled multicontig draft assemblies were generated running Velvet optimizer and Velvet (Zerbino and Birney 2008). Contigs with <300 bases were removed, and scaffolding software SSPACE (Boetzer et al. 2011) was employed. The assembly was further improved using GapFiller (Boetzer and Pirovano 2012). Antimicrobial susceptibility testing was determined by the BSAC agar dilution method (Reynolds et al. 2008). Clustering of regions was assessed on a phylogeny of English EMRSA-15 isolates by counting the proportion of internal nodes whose descendants were isolated from a single region. In order to compare this proportion with the proportions expected when isolates would be distributed randomly, we randomized the leaves 1000 times and calculated the proportions accordingly.

## Data access

All sequences from this study have been submitted to the European Nucleotide Archive (ENA; http://www.ebi.ac.uk/ena) under the study number ERP001012 and individual accession numbers are given in Supplemental Table S2.

## Competing interest statement

All authors declare that they have no conflicts of interest. M.E.T. and J.P. have received support for travel and conference expenses from Illumina. M.T.G.H. is a consultant for Pfizer.

## Supplementary Material

Supplemental Material
