# Supplementary material for: Building a genomic framework for prospective MRSA surveillance in the United Kingdom and the Republic of Ireland
Source: Genome Res. 2016 Feb;26(2):263–70. doi: 10.1101/gr.196709.115 (PMC4728378; doi:10.1101/gr.196709.115)
Supplement: Supplemental Material [file supp_26_2_263__index.html]

Building a genomic framework for prospective MRSA surveillance in the United Kingdom and the Republic of Ireland — Building a genomic framework for prospective MRSA surveillance in the United Kingdom and the Republic of Ireland — Building a genomic framework for prospective MRSA surveillance in the United Kingdom and the Republic of Ireland — Supplemental Material 

# Building a genomic framework for prospective MRSA surveillance in the United Kingdom and the Republic of Ireland

## Supplemental Material

**Files in this Data Supplement:**

- Supplemental Figures S1-S4.pdf
- Supplemental Table S1.pdf
- Supplemental Table S2.xlsx
