# Supplementary material for: Building a genomic framework for prospective MRSA surveillance in the United Kingdom and the Republic of Ireland
Source: Genome Res. 2016 Feb;26(2):263–70. doi: 10.1101/gr.196709.115 (PMC4728378; doi:10.1101/gr.196709.115)
Supplement: Supplemental Material [file supp_gr.196709.115_Supplemental_Figures_S1-4.pdf]

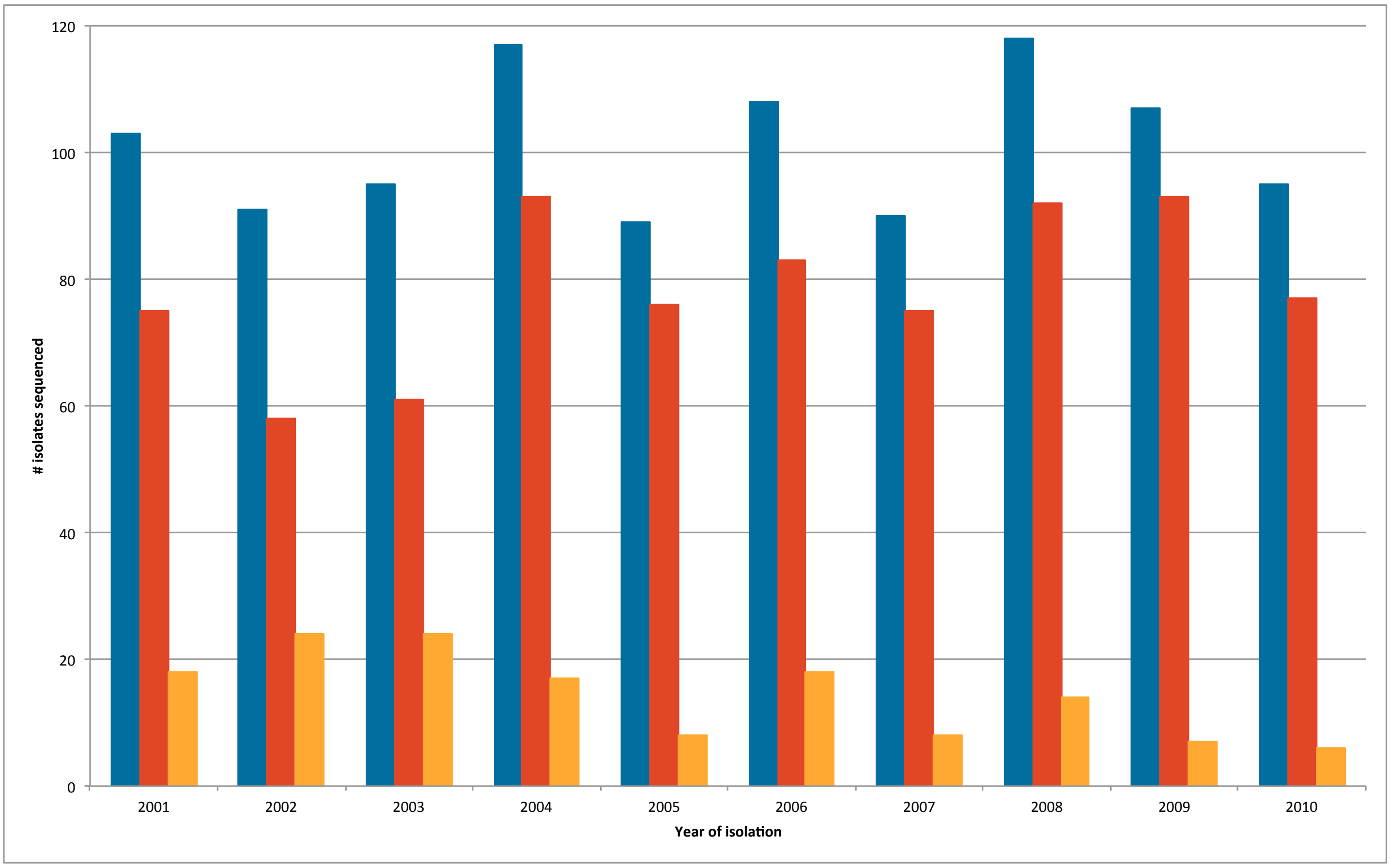

Figure S1. Number of MRSA isolates per year of collection
Total number of isolates – blue, CC22 – red, CC30 – orange.

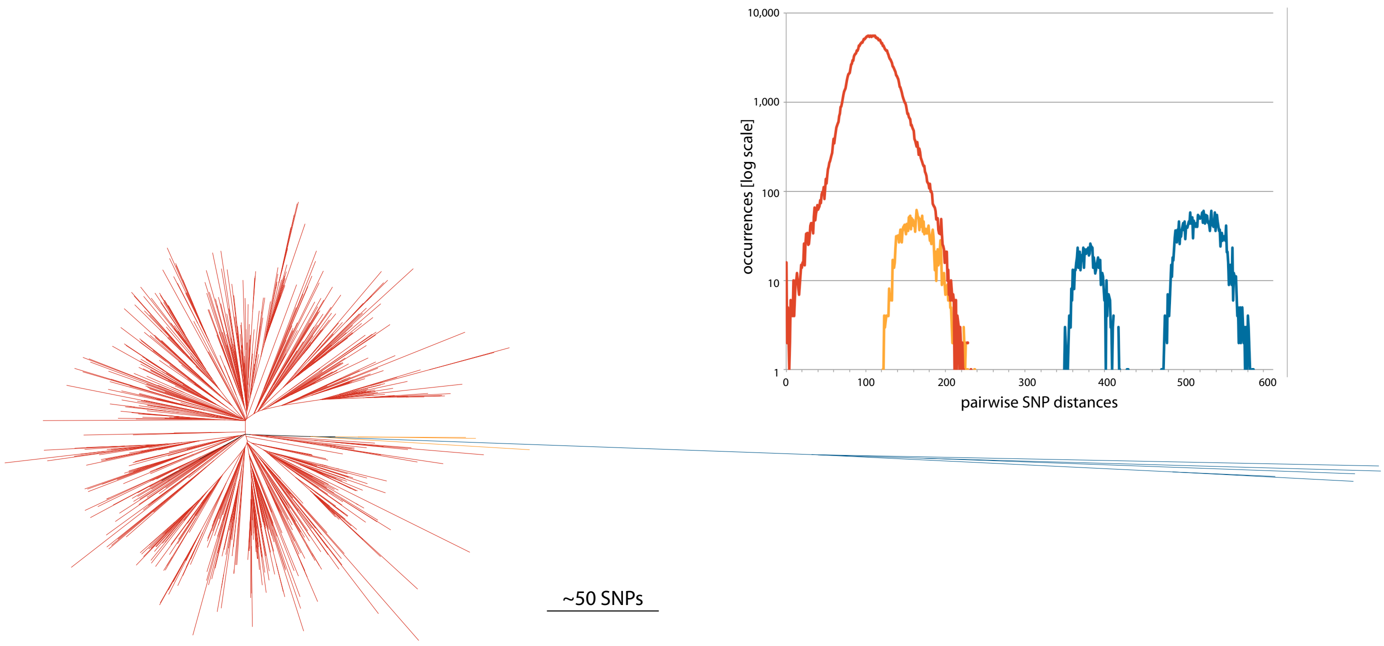

Figure S2. Population structure of CC22 and pairwise SNP distances

Maximum likelihood tree based on 21,848 core SNPs identified by mapping to the reference genome HO 5095 0412. The fluoroquinolone resistant epidemic EMRSA-15 lineage (ST22-A2), as defined by Holden et al, 2013) is shown in red, the fluoroquinolone sensitive progenitor MRSA lineage (ST22-A1) is shown in orange, and community-associated ST22 population (non-ST22-A) are shown in blue. In the inset, the pairwise SNP distances are plotted in log scale within isolates of ST22-A2 (red), between ST22-A2 and ST22-A1 (orange), as well as between ST22-A2 and community isolates (blue).

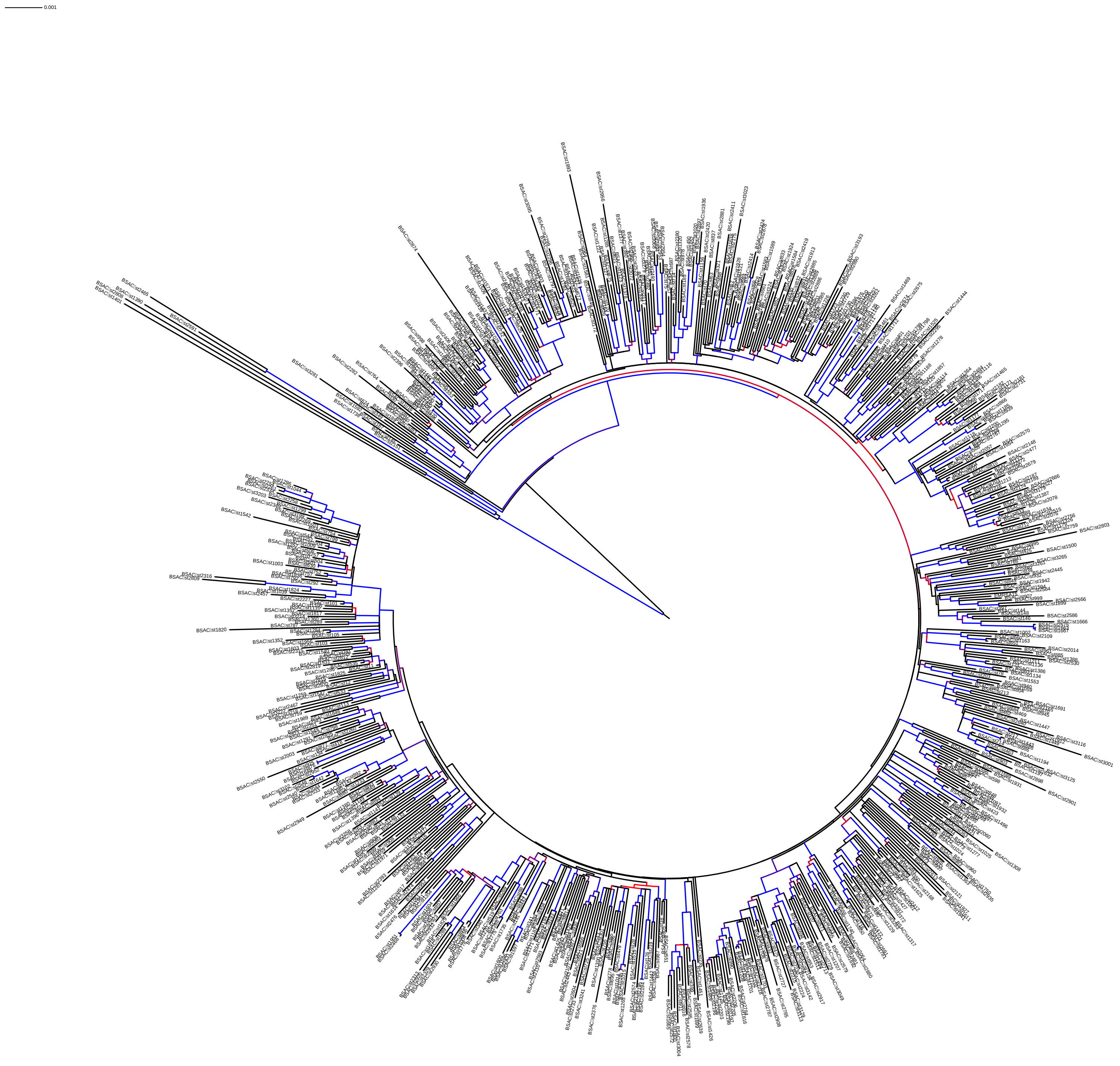

Figure S3. Population structure of CC22 with bootstrap values

Maximum likelihood tree based on 21,848 core SNPs identified by mapping to the reference genome HO 5095 0412. The scale bar represents 0.001 substitutions in core SNPs identified. Nodes with less than 80% bootstrap support are coloured in red, with the maximum bootstrap support of 100% coloured in blue, and values in between coloured in intermediate colours.

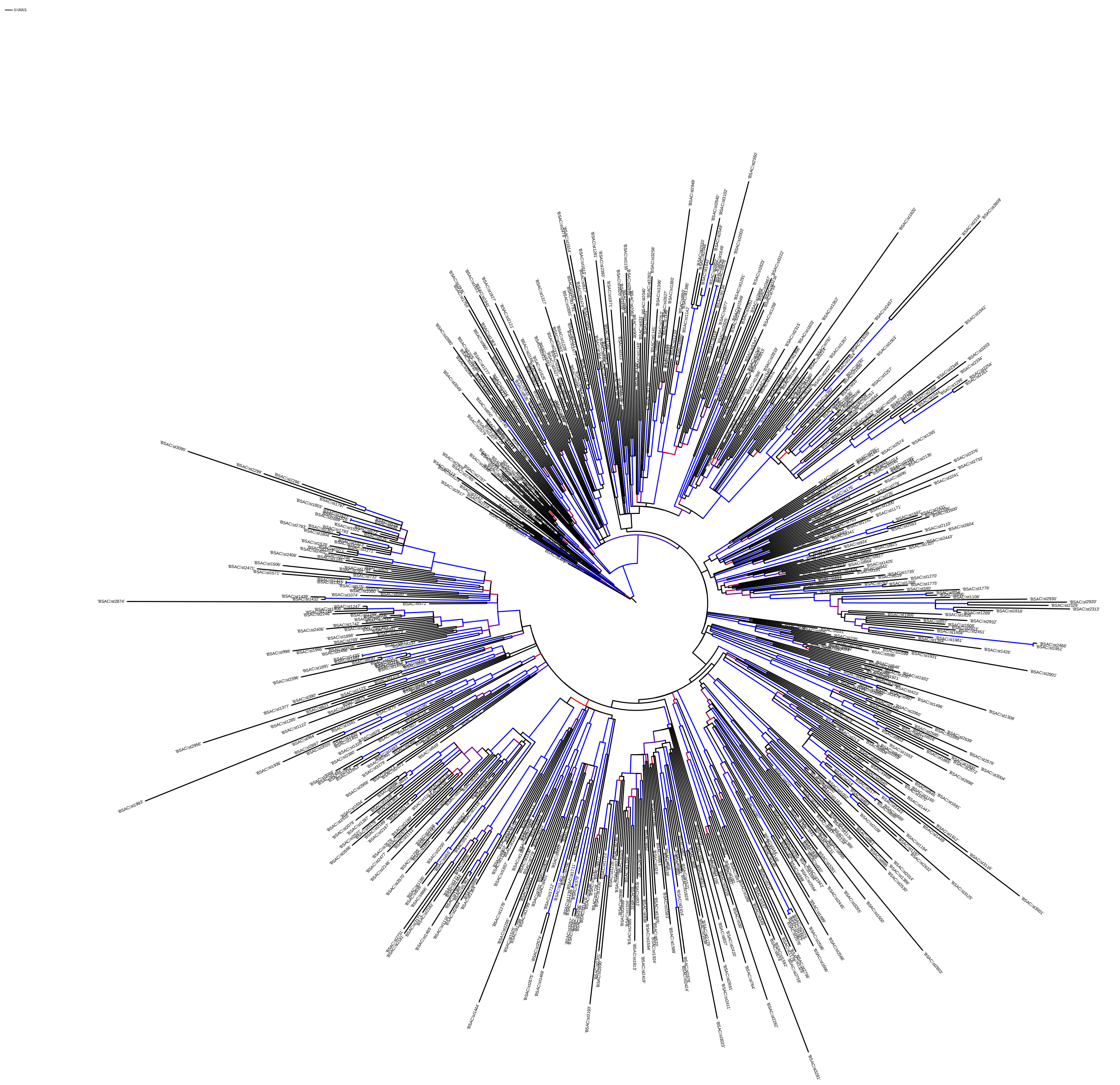

Figure S4. Phylogeny of EMRSA-15

The maximum likelihood tree of ST22-A isolates was based on core SNPs identified by mapping to the reference ST22 genome HO 5095 0412. The total number of SNP sites present in the core genome alignment in this lineage was 20,488 SNPs with the scale bar representing 0.0001 substitutions per core SNPs identified. Nodes with less than 80% bootstrap support are coloured in red, with the maximum bootstrap support of 100% coloured in blue, and values in between coloured in intermediate colours.
