# Supplementary material for: Building a genomic framework for prospective MRSA surveillance in the United Kingdom and the Republic of Ireland
Source: Genome Res. 2016 Feb;26(2):263–70. doi: 10.1101/gr.196709.115 (PMC4728378; doi:10.1101/gr.196709.115)
Supplement: Supplemental Material [file supp_gr.196709.115_Supplemental_Table_S1.pdf]

Table S1. Number of MRSA isolates in each region per year

Target collection (ANY *S. aureus*)	10 *S. aureus*	10 *S. aureus*	10 *S. aureus*	10 *S. aureus*	10 *S. aureus*	10 *S. aureus*	10 *S. aureus*	20 *S. aureus*	20 *S. aureus*	14 *S. aureus*	
**Year**	**2001**	**2002**	**2003**	**2004**	**2005**	**2006**	**2007**	**2008**	**2009**	**2010**	**total number of MRSA isolates per region**
**ENGLAND**											
Central-East Midlands	4 (1)	3 (1)	0 (1)	7 (1)	3 (1)	3 (1)	3 (1)	5 (1)	2 (1)	3 (1)	**33**
East 1	10 (2)	3 (1)	1 (1)	4 (1)	1 (1)	5 (1)	3 (1)	5 (1)	- (0)	8 (2)	**40**
East 2	5 (1)	12 (2)	9 (2)	12 (2)	9 (2)	9 (2)	6 (2)	7 (2)*	8 (2)*	2 (2)	**79**
North-Central	4 (1)	9 (2)	7 (2)	9 (2)	8 (2)	11 (2)	6 (2)	9 (2)	10 (2)	4 (2)*	**77**
North-East 1	3 (1)	2 (1)	3 (1)	5 (1)	4 (1)	4 (1)	1 (1)	4 (1)	4 (1)	0 (1)*	**30**
North-East 2	- (0)	- (0)	- (0)	- (0)	- (0)	- (0)	- (0)	- (0)	- (0)	3 (1)	**3**
North-West 1	4 (1)	6 (1)	4 (1)	6 (1)	2 (1)	1 (1)	7 (1)	6 (1)	4 (1)	3 (2)	**43**
North-West 2	4 (1)	2 (1)	4 (1)	1 (1)	3 (1)	0 (1)*	6 (1)	4 (1)	9 (1)	1 (1)	**34**
South-Central	7 (1)	4 (1)	5 (1)	2 (1)	3 (1)	8 (1)	4 (1)	4 (1)	4 (1)	2 (1)	**43**
South-East	7 (2)	9 (2)	10 (2)	10 (2)	11 (2)	8 (2)*	8 (2)	8 (2)*	7 (3)	9 (4)	**87**
South-West	10 (2)	5 (2)	8 (2)	8 (2)	10 (2)	9 (2)	8 (2)	15 (2)	7 (2)	6 (4)**	**86**
West Midlands	11 (3)	13 (3)	16 (3)	15 (3)	11 (3)	12 (3)	8 (3)	8 (3)	7 (3)*	2 (3)	**103**
**NORTHERN IRELAND**	4 (2)	5 (2)	9 (2)	9 (2)	6 (2)	9 (2)	7 (2)	3 (1)	11 (2)	9 (3)	**72**
**SCOTLAND**	10 (2)	5 (2)	7 (2)*	9 (2)	8 (2)	9 (2)	5 (2)	10 (2)	11 (2)	16 (4)*	**90**
**WALES**	11 (2)	10 (2)	9 (2)	11 (2)	6 (2)	9 (2)	11 (2)	15 (2)	13 (2)	14 (3)	**109**
**IRELAND**	9 (2)	3 (2)	3 (2)	9 (2)	4 (2)	11 (2)	7 (2)	15 (2)	10 (2)	13 (4)	**84**
**total number of MRSA isolates per year**	**103 (24)**	**91 (25)**	**95 (25)**	**117 (25)**	**89 (25)**	**108 (25)**	**90 (25)**	**118 (24)**	**107 (25)**	**95 (38)**	**1013**

Numbers in brackets indicate the number of laboratories contributing any *S. aureus*. * indicates that one lab has contributed less than 90% of target samples; ** indicates two labs have contributed less than 90% of target samples
